# Pre-ICU statin therapy reduces 28-day mortality in sepsis-associated brain dysfunction: a propensity-matched analysis of potential neuroprotective mechanisms

**DOI:** 10.3389/fphar.2025.1586372

**Published:** 2025-09-30

**Authors:** Lei Yu, Shan Zou, Sihao Zheng, Jiangtao Deng, Qingshan Zhou, Jun Jin

**Affiliations:** Department of Intensive Care Unit, The University of Hong Kong-Shenzhen Hospital, Shenzhen, China

**Keywords:** sepsis-associated brain dysfunction, statins, neuroinflammation, oxidative stress, survival, intensive care, retrospective cohort study

## Abstract

**Background:**

Sepsis-associated brain dysfunction (SABD) is a severe complication of sepsis characterized by acute cognitive impairment and altered mental status, contributing to increased morbidity and mortality in intensive care units (ICUs). The pathophysiology involves neuroinflammation, oxidative stress, and blood-brain barrier disruption. Despite evidence suggesting potential anti-inflammatory and antioxidative properties of statins, their neuroprotective effects in SABD patients remain poorly characterized.

**Methods:**

This retrospective cohort study utilized the MIMIC-IV database (version 3.1), including adult ICU patients meeting Sepsis-3.0 criteria and diagnosed with SABD, defined as Glasgow Coma Scale (GCS) score <15 or presence of delirium. Patients with preexisting neurological disorders, chronic alcohol/substance abuse, or severe metabolic imbalances were excluded. Pre-ICU statin use was identified through prescription records. Propensity score matching (PSM) at a 1:1 ratio was performed to balance baseline characteristics between pre-ICU statin users (n = 374) and non-users (n = 374). The primary outcome was 28-day all-cause mortality, with secondary outcomes including ICU mortality, in-hospital mortality, and length of stay. Kaplan-Meier survival analysis and Cox proportional hazards regression models were utilized to assess associations between pre-ICU statin use and clinical outcomes.

**Results:**

Among 1,463 eligible patients, 412 (28.2%) received pre-ICU statin therapy. After PSM, baseline characteristics were well-balanced between groups. Kaplan-Meier analysis demonstrated significantly higher 28-day survival rates among statin users (91% vs. 85%; *P* = 0.0051). Cox regression demonstrated that pre-ICU statin use was independently associated with reduced 28-day mortality (HR: 0.604, 95% CI: 0.380–0.960, P = 0.033). Subgroup analyses revealed consistent protective effects in patients aged ≥65 years, males, those requiring vasopressors, and those on mechanical ventilation. Sensitivity analyses confirmed the robustness of these findings. Secondary outcomes showed trends toward reduced ICU mortality and shorter ICU stays in statin users, though these associations did not reach statistical significance after adjustment.

**Conclusion:**

Pre-ICU statin therapy was associated with improved 28-day survival in SABD patients, potentially attributable to anti-inflammatory and antioxidant mechanisms. Despite limitations inherent in its retrospective design, this study suggests that statins may represent a promising therapeutic option for SABD patients. Prospective randomized controlled trials are warranted to validate these findings and optimize treatment protocols for this vulnerable population.

## Introduction

Sepsis-associated brain dysfunction (SABD) represents a significant clinical challenge in critical care medicine, affecting approximately 70% of septic patients and contributing substantially to morbidity and mortality in intensive care units (ICUs) (Mazeraud et al., 2020; Sonneville et al., 2023; Dumbuya et al., 2023; Gofton and Young, 2012). This neurological complication manifests as acute cognitive impairment and altered mental status, ranging from mild confusion to coma, and is associated with prolonged hospitalization, increased mortality rates, and long-term cognitive sequelae among survivors (Fu et al., 2018; Chen et al., 2020). Despite its clinical significance, therapeutic options for SABD remain limited, underscoring the urgent need for targeted therapeutic interventions (Dumbuya et al., 2023).

The pathophysiological mechanisms underlying SABD are complex and multifaceted, involving intricate interactions between systemic inflammation and cerebral function (Manabe and Heneka, 2021; Czempik et al., 2020). Current evidence suggests that neuroinflammation, oxidative stress, blood-brain barrier disruption, and neurotransmitter imbalances play pivotal roles in the development of SABD (Haruwaka et al., 2019; Zhao et al., 2021). Inflammatory mediators, including cytokines and chemokines released during the systemic inflammatory response, can cross the compromised blood-brain barrier, triggering microglial activation and neuronal dysfunction (Manabe and Heneka, 2021; Sonneville et al., 2017). This cascade ultimately leads to the clinical manifestations characteristic of SABD, including impaired consciousness, delirium, and cognitive deficits (Eidelman et al., 1996).

Statins, primarily prescribed for their lipid-lowering properties, have gained attention for their pleiotropic effects extending beyond cholesterol reduction (Jiang et al., 2021; Köhler-Forsberg et al., 2020). These 3-hydroxy-3-methylglutaryl coenzyme A (HMG-CoA) reductase inhibitors possess anti-inflammatory, antioxidant, and endothelial-stabilizing properties that may confer neuroprotection in various neurological conditions (Köhler-Forsberg et al., 2020; Lee et al., 2018; Fracassi et al., 2019). Experimental studies have demonstrated that statins attenuate microglial activation, reduce pro-inflammatory cytokine production, enhance endothelial nitric oxide synthase activity, and mitigate oxidative stress—mechanisms potentially beneficial in mitigating the pathophysiological processes underlying SABD (Tauber et al., 2020; Bagheri et al., 2020; Shen et al., 2025).

Despite promising preclinical data, clinical evidence evaluating the impact of statin therapy on SABD outcomes remains limited (Lee et al., 2018). Several observational studies have suggested potential benefits of statins in sepsis and critical illness, including reduced mortality and improved organ function (Dobesh and Olsen, 2014; Zhang et al., 2024). However, few investigations have specifically examined the relationship between statin use and neurological outcomes in septic patients (Gu et al., 2021). This knowledge gap impedes the development of evidence-based recommendations regarding statin therapy for patients at risk of developing SABD.

The present study aims to address this critical knowledge gap by investigating the association between pre-ICU statin therapy and clinical outcomes in patients with SABD, with a particular focus on 28-day all-cause mortality. Utilizing a large-scale, real-world database, we sought to determine whether pre-existing statin use confers survival benefits in this vulnerable patient population. Our findings may provide valuable insights into the potential neuroprotective effects of statins in sepsis and inform future clinical trials evaluating targeted interventions for SABD.

## Materials and methods

### Study design and data source

This retrospective cohort study utilized data from the Medical Information Mart for Intensive Care IV (MIMIC-IV) database version 3.1. The MIMIC-IV database is an openly accessible electronic health record repository that includes comprehensive, de-identified medical information from over 50,000 patients admitted to intensive care units (ICUs) at the Beth Israel Deaconess Medical Center (Boston, MA, United States) from 2008 to 2022 (Yu et al., 2025). The study protocol was approved by the Institutional Review Board at Beth Israel Deaconess Medical Center, and the requirement for informed consent was waived due to the retrospective nature of the study (Certificate No.: 56161429).

### Study population and data extraction

We included adult patients (aged ≥18 years) admitted to the ICU with sepsis-associated brain dysfunction (SABD). SABD was defined as the presence of either a Glasgow Coma Scale (GCS) score lower than 15 or a diagnosis of delirium in patients meeting Sepsis-3.0 criteria. The use of the Sepsis-3.0 clinical criteria, as opposed to relying solely on administrative diagnostic codes, was a deliberate methodological choice to ensure a standardized case definition across the entire study period and to mitigate potential biases from secular trends in diagnostic practices, including the transition from ICD-9 to ICD-10. The GCS, a standardized tool for consciousness assessment, evaluates eye response, verbal response, and motor response, with scores ranging from 3 to 15. A score below 15 indicates impaired consciousness, suggesting brain dysfunction. Delirium was included as a diagnostic criterion even in patients with GCS of 15, as it represents fluctuating mental status changes with inattention and either disorganized thinking or altered consciousness levels, which are common manifestations of septic encephalopathy.

**FIGURE 1:**
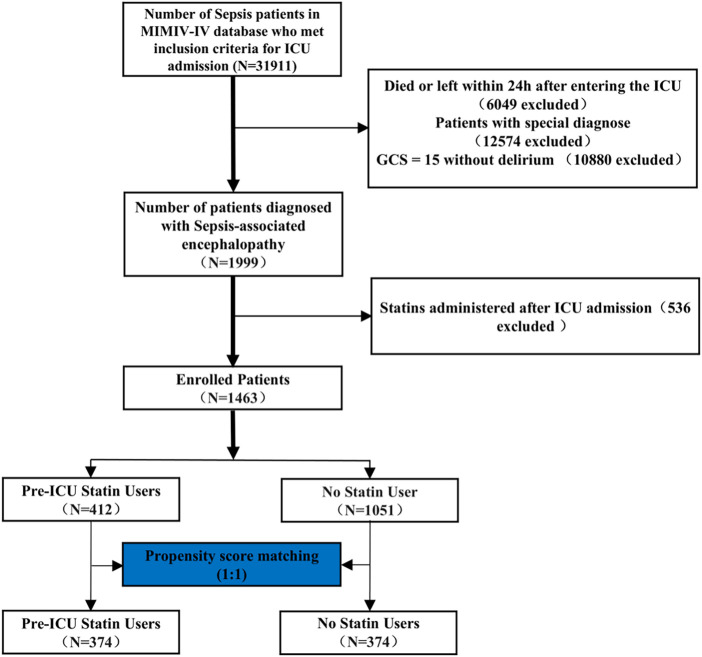
Workflow of the study. GCS: Glasgow Coma Scale. The special diagnostics encompass a range of conditions, including primary neurological injury (such as traumatic brain injury, ischemic stroke, hemorrhagic stroke, epilepsy, or intracranial infection), chronic alcohol or drug abuse, severe electrolyte imbalances (including hyponatremia), hyperglycemia, hypoglycemia, pre-existing liver or kidney failure affecting consciousness, recent cardiac resuscitation, and iron-deficiency anemia.

Inclusion criteria were: (1) age ≥18 years; (2) meeting Sepsis-3.0 diagnostic criteria; (3) ICU stay >24 h; (4) first ICU admission; and (5) complete initial patient information. Exclusion criteria comprised: (1) preexisting neurological conditions (including traumatic brain injury, meningitis, encephalitis, cerebral hemorrhage, cerebral embolism, ischemic stroke, epilepsy, brain tumors, or intracranial infections); (2) psychiatric or neurological disorders; (3) chronic alcohol abuse or substance use disorder; (4) metabolic, hepatic, or hypertensive encephalopathy; (5) severe electrolyte imbalances (sodium <120 mmol/L) or glucose disorders (glucose >180 mg/dL or <54 mg/dL); and (6) missing GCS evaluation. The patient selection process is illustrated in Figure 1.

### Exposure and outcomes

The exposure variable was defined as pre-ICU statin therapy, documented in patients’ prescription records without time restrictions prior to ICU admission. Statin use was ascertained from the prescriptions database and encompassed all routinely prescribed agents—atorvastatin, pravastatin, rosuvastatin, simvastatin, and lovastatin. Patients with indeterminate statin exposure status were excluded from the analysis. The primary endpoint was defined as 28-day all-cause mortality. Secondary endpoints included ICU mortality, in-hospital mortality, ICU length of stay, and hospital length of stay.

### Data collection

Data extraction was performed using Structured Query Language (SQL) with scripts obtained from the GitHub repository (https://github.com/MIT-LCP/mimic-iv). Using PostgreSQL tools (version 16.0), we extracted meaningful clinical data from the MIMIC-IV database, including demographics, such as age, sex, race and ethnicity, and body mass index (BMI); comorbidities, such as hypertension, diabetes mellitus, cancer, heart failure, myocardial infarction, and chronic obstructive pulmonary disease; clinical parameters, including heart rate, mean arterial pressure, respiratory rate, oxygen saturation, and temperature; laboratory values, such as white blood cell count, platelet count, hemoglobin, sodium, potassium, chloride, glucose, lactate, and creatinine; therapeutic interventions, such as vasopressor use, continuous renal replacement therapy, and mechanical ventilation; severity scores, including the Sequential Organ Failure Assessment (SOFA), Acute Physiology Score III (APSIII), Simplified Acute Physiology Score II (SAPSII), and Glasgow Coma Scale (GCS); and the Charlson Comorbidity Index, which measures comorbidities based on administrative data.

### Propensity score matching (PSM)

To minimize selection bias and balance baseline characteristics between statin users and non-users, we conducted propensity score matching (PSM) at a 1:1 ratio. The propensity score was calculated using a logistic regression model that included variables such as age, sex, race, body mass index (BMI), vasopressor use, continuous renal replacement therapy (CRRT), mechanical ventilation (MV), Sequential Organ Failure Assessment (SOFA) score, Acute Physiology and Chronic Health Evaluation III (APACHE III) score, Simplified Acute Physiology Score II (SAPS II), Charlson Comorbidity Index, and Glasgow Coma Scale (GCS). Matching was performed using the nearest neighbor method with a caliper width of 0.2, without replacement. The balance of baseline variables between the pre-ICU statin use and no statin use groups was assessed using standardized mean differences (SMD), with a value less than 0.10 indicating an acceptable balance.

### Statistical analysis

Variables with more than 30% missing values were excluded from the analysis. For variables with less than 30% missing values, multiple imputation was performed to handle the missing data, generating multiple datasets based on the distribution of observed data. Each imputed dataset was analyzed separately, and the results were pooled to yield more accurate and robust parameter estimates (Supplementary Figure S1). Continuous variables were presented as either mean (standard deviation) or median (interquartile range), depending on their distribution, while categorical variables were reported as counts (percentages). Differences between pre-ICU statin users and non-users were evaluated using appropriate statistical tests including t-test, Mann-Whitney U test, chi-square test, or Fisher’s exact test.

To visualize survival probabilities, Kaplan-Meier curves were constructed, and the log-rank test was employed to compare survival differences between pre-ICU statin users and non-users. To evaluate the association between pre-ICU statin use and 28-day all-cause mortality, we constructed Cox proportional hazards regression models. The initial model was unadjusted, followed by a multivariable model that accounted for covariates with a variance inflation factor (VIF) of less than 5, thereby addressing potential confounding factors (Supplementary Figure S2). Secondary outcomes, such as ICU mortality and in-hospital mortality, were assessed using logistic regression models to compute odds ratios (ORs) with 95% confidence intervals (CIs).

All statistical analyses were conducted using R software (version 4.2.3), and a two-tailed P-value of <0.05 was considered statistically significant.

### Sensitivity analyses

Sensitivity analyses were performed to evaluate the robustness of the findings. These analyses were conducted using both the pre-propensity score matching (PSM) and post-PSM cohorts. All covariates with a variance inflation factor (VIF) less than 5 were included in the models to ensure that the results were not driven by multicollinearity or the exclusion of important variables. The aim was to confirm that the association between statin use and 28-day all-cause mortality remained consistent across different analytical approaches and datasets.

### Subgroup analyses

To thoroughly investigate the potential variability in the relationship between pre-ICU statin use and 28-day mortality, we conducted comprehensive subgroup analyses across critical clinical and demographic categories. These included mechanical ventilation status (ventilated vs. non-ventilated), the need for continuous renal replacement therapy (CRRT) (required vs. not required), vasopressor use (used vs. not used), body mass index (BMI ≥30 kg/m^2^ vs. <30 kg/m^2^), sex (male vs. female), and age groups (≥65 years vs. <65 years). Hazard ratios (HR) and corresponding 95% confidence intervals (CIs) were calculated for each subgroup using Cox proportional hazards regression models. Additionally, we formally tested for interaction effects between pre-ICU statin use and these categorical covariates by incorporating interaction terms into the regression models, thereby enabling a precise assessment of subgroup-specific associations.

## Result

### Patient selection

Figure 1 illustrates the process of patient selection. Of 31,911 sepsis patients from the MIMIC-IV database, 1,999 were diagnosed with sepsis-associated encephalopathy. After excluding patients with special diagnoses (including primary neurological injury, chronic alcohol/drug abuse, severe electrolyte imbalances, pre-existing liver or kidney failure, recent cardiac resuscitation, and iron-deficiency anemia), those who received statins post-ICU admission, and applying propensity score matching (1:1), 374 pre-ICU statin users and 374 non-statin users were included in the final analysis. The details of the ICD codes for these diagnoses are provided in Supplementary Table S1.

### Patient characteristics

Of 1463 eligible patients, 412 (28.2%) received pre-ICU statin therapy. Before propensity score matching, patients with pre-ICU statin therapy were older (median [IQR] age, 72.0 [63.0–80.0] vs. 64.0 [51.0–76.0] years) and more likely to be male (64.1% vs. 50.2%). They had a higher prevalence of cardiovascular comorbidities, including hypertension (69.2% vs. 43.3%), diabetes mellitus (30.1% vs. 14.3%), congestive heart failure (28.6% vs. 16.3%), and previous myocardial infarction (9.0% vs. 0.9%). Propensity score matching yielded 374 matched pairs. In the matched cohort, most baseline characteristics were well balanced between groups, although some differences in cardiovascular comorbidities remained. The matched groups showed similar severity of illness, as indicated by comparable SOFA, APSIII, and SAPSII scores, as well as Glasgow Coma Scale scores(Table 1). The distributional balance before and after propensity score matching and variance inflation factor analysis for potential confounders are presented in the Supplementary Figure S3.

**TABLE 1:** Baseline characteristics before and after propensity score matching.

Variables	Before propensity score matching			After propensity score matching		
	No statin use	Pre-ICU statin use	SMD	No statin use	Pre-ICU statin use	SMD
	N = 1051	N = 412		N = 374	N = 374	
Age (yr), median (IQR)	64.00 (51.00, 77.00)	72.00 (64.00, 80.00)	0.611	74.00 (62.00, 84.00)	72.00 (64.00, 79.00)	0.037
Gender (%)						
Male	528 (50.2%)	264 (64.1%)	0.282	318 (85.0%)	342 (91.4%)	0.016
Female	523 (49.8%)	148 (35.9%)		56 (15.0%)	32 (8.6%)	
Race (%)						
White	711 (67.6%)	303 (73.5%)	0.237	269 (71.9%)	276 (73.8%)	0.054
Black	74 (7.0%)	15 (3.6%)		16 (4.3%)	14 (3.7%)	
Asia	44 (4.2%)	6 (1.5%)		5 (1.3%)	6 (1.6%)	
Hispanic	33 (3.1%)	10 (2.4%)		9 (2.4%)	8 (2.1%)	
Others	189 (18.0%)	78 (18.9%)		75 (20.1%)	70 (18.7%)	
BMI (kg m-2), median (IQR)	27.06 (23.42, 32.39)	28.78 (25.41, 32.81)	0.137	27.68 (23.71, 32.69)	28.66 (25.31, 32.79)	0.058
Comorbidity						
Hypertension (%)						
No	596 (56.7%)	127 (30.8%)	0.54	174 (46.5%)	115 (30.7%)	0.328
Yes	455 (43.3%)	285 (69.2%)		200 (53.5%)	259 (69.3%)	
Diabetes Mellitus (%)						
No	901 (85.7%)	288 (69.9%)	0.388	311 (83.2%)	265 (70.9%)	0.295
Yes	150 (14.3%)	124 (30.1%)		63 (16.8%)	109 (29.1%)	
Malignant tumor (%)						
No	866 (82.4%)	321 (77.9%)	0.113	302 (80.7%)	295 (78.9%)	0.047
Yes	185 (17.6%)	91 (22.1%)		72 (19.3%)	79 (21.1%)	
Congestive heart failure (%)						
No	880 (83.7%)	294 (71.4%)	0.3	286 (76.5%)	268 (71.7%)	0.11
Yes	171 (16.3%)	118 (28.6%)		88 (23.5%)	106 (28.3%)	
Myocardial Infarction (%)						
No	1042 (99.1%)	375 (91.0%)	0.382	370 (98.9%)	340 (90.9%)	0.372
Yes	9 (0.9%)	37 (9.0%)		4 (1.1%)	34 (9.1%)	
COPD (%)						
No	1000 (95.1%)	396 (96.1%)	0.047	350 (93.6%)	360 (96.3%)	0.122
Yes	51 (4.9%)	16 (3.9%)		24 (6.4%)	14 (3.7%)	
Vital Sign, median (IQR)						
Heart rate (beats min-1)	90.00 (77.00, 106.00)	82.00 (76.00, 90.75)	0.376	86.00 (76.00, 98.25)	82.00 (76.00, 91.00)	0.173
MBP (mmHg)	79.00 (69.00, 91.00)	73.00 (65.00, 81.00)	0.344	77.00 (68.00, 89.00)	73.00 (65.00, 81.00)	0.301
RR (min-1)	19.00 (15.00, 24.00)	16.00 (14.00, 19.00)	0.533	18.00 (15.00, 22.00)	16.00 (14.00, 19.25)	0.404
SpO2(%)	98.00 (95.00, 100.00)	100.00 (98.00, 100.00)	0.361	99.00 (95.00, 100.00)	100.00 (97.00, 100.00)	0.309
Temperature (°C)	36.78 (36.44, 37.11)	36.67 (36.33, 37.00)	0.203	36.67 (36.39, 37.06)	36.67 (36.33, 37.00)	0.05
Laboratory tests, median (IQR)						
WBC (103/uL)	13.91 (8.86)	13.06 (7.07)	0.107	12.30 (8.67, 16.20)	11.95 (8.60, 15.90)	0.033
Platelet (103/uL)	227.68 (113.77)	188.27 (92.86)	0.38	207.00 (140.75, 270.00)	171.50 (129.75, 231.75)	0.282
Hemoglobin (g/dL)	10.91 (2.23)	10.10 (1.95)	0.384	10.85 (9.47, 12.40)	10.00 (8.60, 11.50)	0.392
Sodium (mmol/L)	138.70 (4.27)	138.58 (4.00)	0.03	139.00 (137.00, 141.00)	139.00 (137.00, 141.00)	0.048
Potassium (mmol/L)	4.17 (0.73)	4.19 (0.58)	0.033	4.10 (3.70, 4.50)	4.20 (3.80, 4.50)	0.043
Chloride (mmol/L)	104.90 (5.79)	107.01 (5.50)	0.373	105.00 (102.00, 109.00)	108.00 (104.00, 111.00)	0.321
Glucose (mg/dL)	143.94 (16.08)	141.99 (15.43)	0.124	143.00 (132.00, 157.00)	139.00 (129.00, 154.00)	0.169
Lactate (mmol/L)	2.06 (1.33)	2.07 (1.03)	0.012	1.70 (1.30, 2.50)	1.80 (1.30, 2.60)	0.019
Creatinine (mg/dL)	1.15 (1.51)	1.04 (0.74)	0.096	0.90 (0.70, 1.10)	0.90 (0.70, 1.10)	0.039
Treatment at ICU admission (%)						
Vasopressor (%)						
No	649 (61.8%)	154 (37.4%)	0.503	164 (43.9%)	151 (40.4%)	0.07
Yes	402 (38.2%)	258 (62.6%)		210 (56.1%)	223 (59.6%)	
CRRT (%)						
No	1026 (97.6%)	404 (98.1%)	0.03	366 (97.9%)	367 (98.1%)	0.019
Yes	25 (2.4%)	8 (1.9%)		8 (2.1%)	7 (1.9%)	
Ventilation (%)						
No	550 (52.3%)	142 (34.5%)	0.366	143 (38.2%)	136 (36.4%)	0.039
Yes	501 (47.7%)	270 (65.5%)		231 (61.8%)	238 (63.6%)	
Scoring at ICU admission, median (IQR)						
SOFA	5.00 (3.00, 7.00)	5.00 (4.00, 7.00)	0.076	5.00 (3.00, 7.00)	5.00 (4.00, 7.00)	0.039
APSIII	45.00 (33.00, 61.00)	40.50 (31.00, 59.00)	0.13	44.00 (33.00, 57.25)	41.50 (32.00, 60.00)	0.016
SAPSII	37.00 (28.00, 47.00)	39.00 (32.00, 48.75)	0.151	40.00 (33.00, 49.00)	39.00 (32.00, 49.00)	0.049
GCS	14.00 (10.00, 14.00)	14.00 (9.00, 14.00)	0.077	14.00 (9.00, 14.00)	14.00 (10.00, 14.00)	0.014
Charlson Comorbidity Index	4.00 (2.00, 6.00)	4.00 (3.00, 6.00)	0.365	5.00 (3.00, 6.00)	4.00 (3.00, 6.00)	<0.001

For each variable, median (interquartile range), or number (percent) was reported (as appropriate). BMI, body mass index; MBP, mean blood pressure; SpO2, pulse oxygen saturation; WBC, white blood cell; Hb, hemoglobin; Cr, creatinine; CRRT, continuous renal replacement therapy; MI, myocardial infarct; CHF, congestive heart failure; COPD, chronic obstructive pulmonary disease; SOFA, Sequential Organ Failure Assessment APSIII, Acute Physiology Score III, SAPSII, Simplified Acute Physiology Score II; SOFA, sequential organ failure assessment; ICU, intensive care unit.

### Primary outcome

**FIGURE 2:**
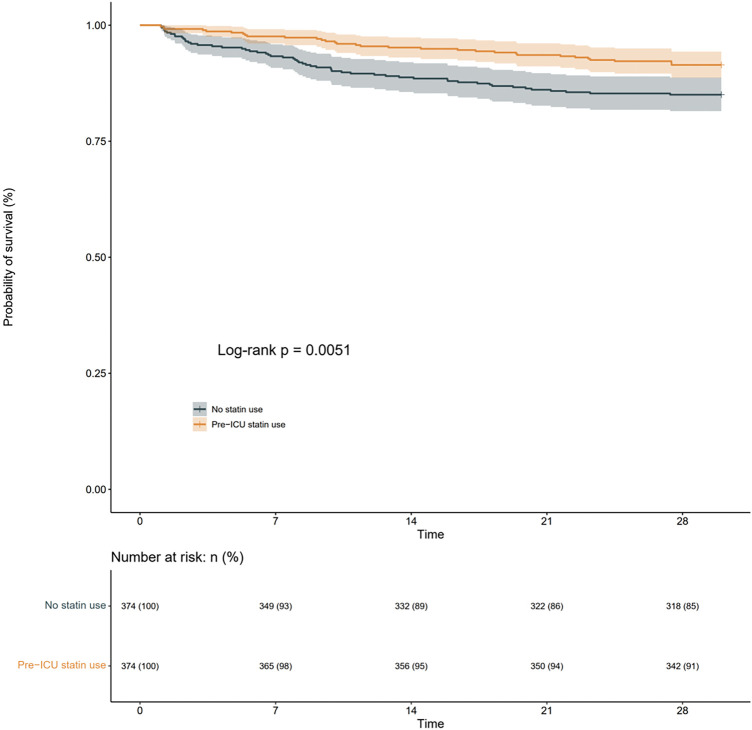
KaplaneMeier curve for 28-day all-cause mortality according to pre-ICU statin users and non-users in the matched cohort.

To investigate the association between pre-ICU statin use and 28-day all-cause mortality in patients with Sepsis-Associated Brain Dysfunction (SABD), we conducted survival analyses using both Kaplan-Meier curves and Cox proportional hazards models. The Kaplan-Meier analysisrevealed that survival curves began to diverge early after ICU admission and maintained a consistent separation throughout the follow-up period (Figure 2). The number at risk showed a gradual decrease in both groups, with pre-ICU statin users maintaining higher survival rates. At day 28, the survival probability was 91% (342/374) for pre-ICU statin users compared to 85% (318/374) for non-users, representing an absolute risk reduction of 6%. The log-rank test demonstrated a statistically significant difference between groups (p = 0.0051), suggesting improved survival associated with pre-ICU statin use (Figure 2).

In the Cox proportional hazards regression analysis, we employed both univariable and multivariable models, with the latter adjusting for covariates having variance inflation factors (VIF) below 5 (Supplementary Figure S2). The univariable analysis showed that pre-ICU statin use was significantly associated with reduced 28-day mortality (HR: 0.543, 95% CI: 0.352–0.838, p = 0.006). This protective association persisted in the multivariable model after adjusting for demographic, clinical, and laboratory variables, with pre-ICU statin use independently predicting lower mortality risk (HR: 0.604, 95% CI: 0.380–0.960, p = 0.033) (Table 2). These findings provide robust evidence that pre-ICU statin use may confer a protective effect against mortality in SABD patients, even after accounting for potential confounding factors.

**TABLE 2:** Association Between Pre-ICU Statin Use and Clinical Outcomes in the matched cohort.

Outcomes	No statin use (N = 374)	Pre-ICU statin use (N = 374)	Univariable analysis		Multivariable analysis^※^	
			HR/OR (95% CI)	P-value	HR/OR (95% CI)	P-value
Primary outcome						
28-day all-cause mortality^a^, (n%)	56 (15.0%)	32 (8.6%)	0.543 (0.352,0.838)	0.006	0.604 (0.380,0.960)	0.033
Secondary outcomes						
ICU mortality^b^, n (%)	25 (6.7%)	14 (3.7%)	0.543 (0.278∼1.062)	0.074	/	/
In-hospital mortality^b^, n (%)	41 (11.0%)	24 (6.4%)	0.557 (0.329∼0.942)	0.029	1.441 (0.618∼3.356)	0.397
Length of ICU stay (days)^b^, median (IQR)	3.42 (1.92, 6.25)	2.22 (1.30, 4.04)	0.971 (0.948∼0.995)	0.018	0.996 (0.976∼1.016)	0.665
Length of hospital stay (days)^b^, median (IQR)	8.71 (5.69, 16.48)	8.21 (5.95, 12.89)	0.997 (0.985∼1.008)	0.544	/	/

CI, confidence interval; HR, hazard ratio; IQR, interquartile range; OR, odds ratio; ※Adjusted for Age, Gender, Race, BMI, hypertension, Diabetes Mellitus, Malignant tumor, Heart failure, Myocardial Infarction; COPD, heartrate, MBP, RR, SpO2, temperature, White Blood Cell count, Platelet count, Hemoglobin, Sodium, Potassium, Chloride, Glucose, Lactate, Creatinine, Vasopressor, Continuous Renal Replacement Therapy, Ventilation, SOFA, APSIII, GCS, and Charlson Comorbidity Index.

^a^ HR, with 95% CI, was calculated using Cox proportional hazards model.

^b^ OR, with 95% CI, was calculated using logistic regression model.

### Subgroup analyses

**FIGURE 3:**
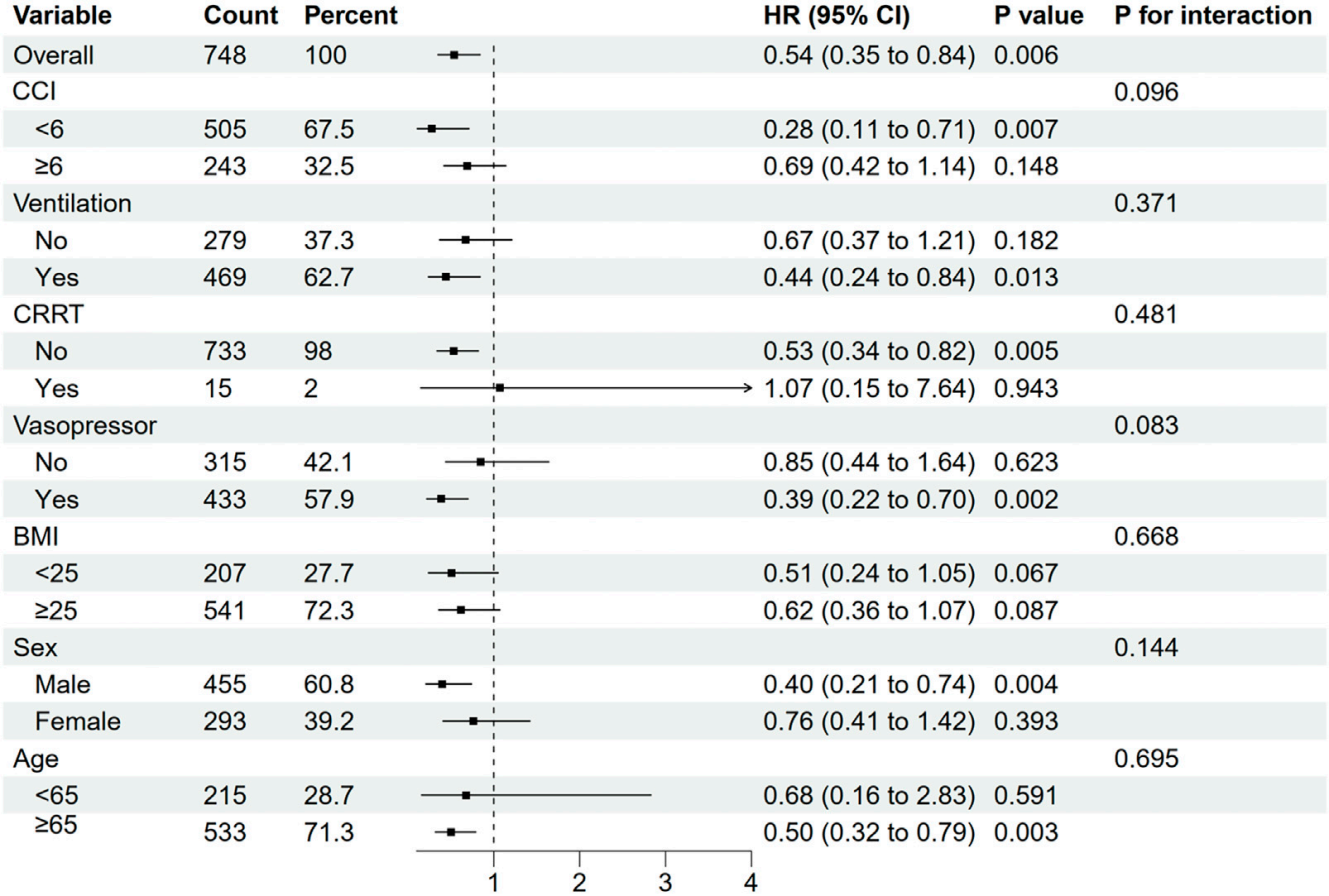
Subgroup analyses for 28-day all-cause mortality in the matched cohort. CCI,Charlson Comorbidity Index; CRRT, continuous renal replacement therapy; BMI, body mass index; CI, confidence interval; HR, hazard ratio.

Subgroup analyses were performed to evaluate whether the association between pre-ICU statin use and mortality varied across different patient characteristics. In the subgroup analyses, significant associations were observed in patients with lower Charlson Comorbidity Index (CCI <6: HR: 0.28, 95% CI: 0.11–0.71, p = 0.007), those requiring vasopressor support (HR: 0.39, 95% CI: 0.22–0.70, p = 0.002), male patients (HR: 0.40, 95% CI: 0.21–0.74, p = 0.004), patients aged ≥65 years (HR: 0.50, 95% CI: 0.32–0.79, p = 0.003), and those requiring ventilation (HR: 0.44, 95% CI: 0.24–0.84, p = 0.013) (Figure 3). However, the effect was not significant in their corresponding counterpart groups (CCI ≥6: HR: 0.69, 95% CI: 0.42–1.14, p = 0.148; no vasopressor: HR: 0.85, 95% CI: 0.44–1.64, p = 0.623; female: HR: 0.76, 95% CI: 0.41–1.42, p = 0.393; age <65: HR: 0.68, 95% CI: 0.16–2.83, p = 0.591; no ventilation: HR: 0.67, 95% CI: 0.37–1.21, p = 0.182) (Figure 3). Tests for interaction showed no significant differences in the effect of statin use between any of the subgroup pairs (all p for interaction >0.05), suggesting that the observed variations in statistical significance between subgroups should be interpreted with caution and may be due to differences in sample size or event rates rather than true differential effects (Figure 3).

### Sensitivity analyses

Sensitivity analysis using the original cohort (n = 1,463) before propensity score matching demonstrated consistent findings. In this larger cohort, pre-ICU statin use maintained its significant association with lower 28-day mortality in both univariable (HR: 0.550, 95% CI: 0.379–0.798, p = 0.002) and multivariable analyses (HR: 0.584, 95% CI: 0.393–0.867, p = 0.008). These results from the unmatched population further supported our primary findings from the propensity score-matched analysis, reinforcing the robustness of the association between pre-ICU statin use and improved survival in SABD patients (detailed results including Kaplan-Meier survival curves and Cox regression analyses for the original cohort are provided in Supplementary Figure S2.

### Secondary outcomes

For secondary outcomes, in the matched cohort, pre-ICU statin use was associated with lower in-hospital mortality (6.4% vs. 11.0%, OR: 0.557, 95% CI: 0.329–0.942, p = 0.029) and shorter ICU length of stay (2.22 vs. 3.42 days, OR: 0.971, 95% CI: 0.948–0.995, p = 0.018) in univariable analysis. However, these associations were not maintained after adjusting for confounders in multivariable analysis (in-hospital mortality: OR: 1.441, 95% CI: 0.618–3.356, p = 0.397; ICU length of stay: OR: 0.996, 95% CI: 0.976–1.016, p = 0.665). ICU mortality showed a trend towards reduction in the statin group (3.7% vs. 6.7%, OR: 0.543, 95% CI: 0.278–1.062, p = 0.074), while hospital length of stay was comparable between groups (8.21 vs. 8.71 days, OR: 0.997, 95% CI: 0.985–1.008, p = 0.544) (Table 2). Similar patterns were observed in the original cohort before matching, with detailed results provided in Supplementary Table S2.

## Discussion

In this large retrospective cohort study utilizing the MIMIC-IV database, we found that pre-ICU statin use was independently associated with reduced 28-day mortality in patients with sepsis-associated brain dysfunction. This protective effect persisted after propensity score matching and multivariable adjustment, with a hazard ratio of 0.604 (95% CI: 0.380–0.960). Importantly, the beneficial association was consistent across various subgroups, particularly pronounced in patients with high comorbidity burden, those requiring vasopressor support, and younger patients (<65 years).

These findings expand current evidence on the pleiotropic benefits of statins in critical illness (Van de Louw et al., 2021; Yao et al., 2024; Yang et al., 2024). While previous studies have examined statin use in sepsis with mixed results (Al-Husinat et al., 2023; Hills et al., 2023; Li et al., 2024; Hashem et al., 2022), our study specifically focused on patients with sepsis-associated brain dysfunction, a particularly vulnerable population with historically poor outcomes (Gofton and Young, 2012; Ren et al., 2020). The observed mortality reduction aligns with meta-analyses suggesting statin benefits in sepsis (Liang et al., 2022), but provides novel evidence for their role in neurological manifestations of sepsis.

Several mechanisms might explain the observed benefits of pre-ICU statin use, Beyond their lipid-lowering effects, statins exhibit pleiotropic properties including anti-inflammatory, antioxidant, and endothelial-protective effects (Liang et al., 2022; Catalão et al., 2020; Tang et al., 2024; Terblanche et al., 2007; Kuebart et al., 2023). In sepsis-associated brain dysfunction, statins may preserve blood-brain barrier integrity, reduce neuroinflammation, and improve cerebral microcirculation (Shen et al., 2025; Catalão et al., 2020; Steckert et al., 2014). The consistent benefit observed across subgroups suggests these mechanisms may be particularly relevant in the context of sepsis-induced neurological dysfunction.

Our findings carry significant clinical implications. The data suggest that pre-existing statin therapy should be continued during ICU admission for patients with sepsis-associated brain dysfunction, absent specific contraindications (Zhang et al., 2024; Mansur et al., 2015). However, our study does not address whether initiating statin therapy during ICU stay would confer similar benefits, highlighting an important area for future research.

The secondary outcomes analysis revealed interesting patterns, with univariable analyses showing reduced in-hospital mortality and shorter ICU length of stay among statin users, although these associations did not persist in multivariable analyses. This suggests that the primary benefit of pre-ICU statin use may be most evident in early mortality reduction, while other factors may have greater influence on hospital course and length of stay (Li et al., 2024; Zhou et al., 2025).

These findings raise several important questions for future research. Prospective randomized trials are needed to definitively establish causality and optimal timing of statin therapy in sepsis-associated brain dysfunction. Studies investigating the impact of different statin types, doses, and duration of therapy would provide valuable guidance for clinical practice. Additionally, research into the biological mechanisms underlying the observed benefits, particularly regarding blood-brain barrier function and neuroinflammation, could identify new therapeutic targets.

Our study has several strengths, including its large sample size, rigorous propensity score matching, and comprehensive sensitivity analyses. The use of the MIMIC-IV database provided detailed clinical information allowing for thorough adjustment for potential confounders. However, several important limitations must be acknowledged. Foremost among these are constraints related to the granularity of medication data. While the MIMIC-IV database identifies the specific type of statin used, we made a deliberate methodological decision to group all agents into a single class to preserve statistical power. Consequently, our study robustly addresses the effect of “any” statin use but cannot comment on the potential differential effects of individual agents. Furthermore, the database lacks detailed information on the dosage or duration of pre-admission therapy, which are true unmeasured confounders. Additionally, this study is subject to limitations inherent to registry analyses, most notably missing data. We observed that some covariates had missingness up to 30%. Formal testing using Little’s test rejected the hypothesis that data were Missing Completely At Random (MCAR), indicating that a Missing At Random (MAR) assumption was more suitable. Based on this finding, we deliberately chose multiple imputation as our primary analytical strategy. A complete-case analysis, which is typically only valid under the strict MCAR assumption, was therefore considered statistically inappropriate for our dataset as it would have risked introducing significant selection bias while substantially reducing statistical power. While multiple imputation is the recommended and more robust method under the MAR assumption, we acknowledge that if the underlying mechanism were Missing-Not-At-Random (MNAR)—a possibility that cannot be definitively tested—residual bias could persist. Finally, while this retrospective design was mitigated by rigorous propensity score matching, it inherently cannot definitively establish causality. The exclusion of patients with certain pre-existing neurological conditions also defines the boundaries of our conclusions. Our findings are based on a “dry lab” (computational) analysis, meaning they establish a clinical association but do not investigate the underlying biological mechanisms. This particular limitation points directly to a crucial future direction: “wet lab” validation through laboratory-based experiments. These limitations collectively underscore the clear necessity for future prospective and mechanistic studies.

## Conclusion

Our study provides compelling evidence supporting the association between pre-ICU statin use and reduced mortality in patients with sepsis-associated brain dysfunction. The significant survival advantage observed suggests that statin therapy may represent a viable therapeutic strategy for this vulnerable group. However, further investigations are essential to elucidate the underlying mechanisms and to refine treatment protocols, ultimately aiming to improve patient outcomes in critical care settings.

## Data Availability

The original contributions presented in the study are included in the article/Supplementary Material, further inquiries can be directed to the corresponding authors.
