# Supplementary material for: Pre-ICU statin therapy reduces 28-day mortality in sepsis-associated brain dysfunction: a propensity-matched analysis of potential neuroprotective mechanisms
Source: Front Pharmacol. 2025 Sep 30;16:1586372. doi: 10.3389/fphar.2025.1586372 (PMC12517583; doi:10.3389/fphar.2025.1586372)
Supplement: Supplementary file 1 [file Table1.docx]

Table s1:The details of the ICD codes for these diagnoses

| Diagnosis | ICD-10 |
| --- | --- |
| Sepsis | 31911 |
| Traumatic Brain Injury | Z87820,V1552,S062X9A,S062X0A,S06300A,S06309A,S062X7A,S062X1A,S062X9S,S06300S,S062X6A,S06300D,S06301A,S06309S,S062X9D,S062X0D,Z13850 |
| Post - traumatic Stress Disorder(PTSD) | F4310,30981 |
| Meningitis | 3682,490,3689,3229,9181,G030,A879,3643,G009,G039,470,3209,3207,G038,3203,3210,B003,7816,3202,G003,3220,R291,32082,B021,G031,3222,G008,G001,G032,G002,A6921,3201,B010,32089,A870,3221,D8681,A878,9042,9882,A390,A392,Z2231,A872,32081,A170,3212,G000,11283,A3211,A5481,A393,A394 |
| Encephalitis | 462,1363,5821,G0481,5829,632,3239,G0490,1361,A86,B004,32381,B941,B020,B582,32362,G053,B1009,9041,32301,G042,32341,G0400,G0401,A1782,A858,A9231,B0111,A849,630,A832,A848,A852,L0882 |
| Cerebral Infarction | Z8673,V1254,43310,43491,I69354,43411,I69351,I639,I69320,I63412,I6340,I63411,I69392,I69322,I69391,43311,I69328,I69311,I69319,I63512,I6349,I63511,I63432,43401,43331,I63431,I63442,I63231,I63441,I63132,I63131,I69334,I63312,I63311,I63232,I63443,43321,I69331,I69393,I69344,I6310,I69312,I6930,I63422,I6319,43301,I69341,I63421,I6302,I63032,I63532,I63531,I63031,I6931,I6312,I69321,I63433,I63522,I63212,I6329,I6350,I63521,I63449,I63211,I6322,I63541,I69365,I69353,I63413,43381,I63233,I69323,I69390,I63542,I69352,I63112,I636,I63133,I63439,I63012,I6359,I63331,I6330,I6339,I63419,I69315,I63423,I63543,I63111,I69314,I63513,I69310,I63523,I69364,I63341,I69369,I63213,I63533,I63113,I63011, I6309,I63332,I63321,I63429,I63013,I63549,I63322,I6300,I69313,I6320,I63239,I63349,I63333,I69333,I63219,I63539,I63139,43391,I63313,I63529,I63343,I63119,I63342 |
| Subarachnoid Hemorrhage(SAH) | 430,S066X9A,S066X0A,85201,I609,85200,85206,I608,85202,S066X1A,I602,S066X7A,I606,I6031,I6012,I6032,I6011,85205,I604,S066X9D,I607,I69098,I69054,S066X6A,S066X0D,S066X9S,I6001,85209,I69092,I69020,S066X8A,85211,I6052,I6051,I6022,85203,I69051,I69028,I6020,I6002,I69021,S066X0S,85204,I6010,I69011,S066X1D,85210,85212,I6900,85216,I69018,I6021,I69022,I69044,I6901,S066X3A |
| Intracerebral Hemorrhage | I618,I611,I615,I619,I610,I614,I69198,I612,I69154,I69151,I613,I69120,I616,S06350A,I69192,I69191,S06360A,I69122,S06369A,I69119,S06351A,I69193,I69128,I69131,I69118,I69112,I69165,I69159,I69111,I6911,I69144,I69134,I69153,I69121,I69141,I69110,I69169,S06357A,S06361A,I69190,I69152,S06360S,I69113,I69132,S06362A,I69164 |
| Epilepsy | 34590,G40909,T426X5A,G40409,34510,34540,34550,G40209,34580,G40109,G40802,T4275XA,G40901,34541,F445,34591,T426X6A,34511,34551,T426X2A,G8384,G40401,Z820,G40919,G40509,34500,G40119,G40419,G40219,64941,34570,G40801,T426X1A,R561,64943,34581,G40101,G40411,34571,G40309,34501,G40804,G40A09,78033,G40911,G40201,G40111,G40009,G40814,G40812,G40B09,G40501,G40211,T4276XA,G40319,G40803,64944,G40311,T4272XA,G40019,T4271XA,G40B19,G40001,G40A19,G40811,T426X5S,G40A01,T425X6A,T426X4A,T425X5A,T426X5D,G40A11,34561,T425X5S |
| Brain Tumor | 2396,D496 |
| Other Cerebrovascular Diseases and Sequelae | 43889,43820,43811,V171,43883,99702,4379,Q282,43813,4387,74781,43882,I6789,I67848,I69954,43819,4389,4380,43822,I97821,4378,43821,I679,I97820,4386,I69998,I69951,43810,Q283,43884,I69898,I69920,4371,I67841,I69854,43812,43853,I69992,43850,I69851,I69991,I69820,I69922,43885,I69928,I69822,I69911,I69891,I69931,I6990,I69934,I69944,I69892,43881,I69828,43830,I69919,I69959,I69811,I688,43814,I69859,I69831,67404,67403,I6991,I69818,I69993,I6781,G468,43831,I69819,I69941,43852,67401,I69834,436,43840,I69841,I6980,I69893,I69921,43832,I69823,I69918,I69965,I69949,I69865,I69844,I69853,I69864,I69833,I69990,I69939,43842,I69810,I69815,I69821,I69912,I69852 |
| Mental Disorders | 2948,O99344,64841,2949,64843,F09,3009,F23,O99343,29189,2939,F068,O99342,O99345,F99,O99341,64844,2929,F5105,29389,29289,V118,F10251,9696,F15959,F12159,F15159,E8541,29382,29381,3108,31089,3109,F15151,64842,F10151,2919,F15150,F062,F10159,F10259,F488,32702,O99340,F14159,F10959,V119,F12259,F11159,E9396,F5113,F11151,F10250,F060,F11951,F15259,F11250,F14150,F11150,32715 |
| Alcohol - related Diseases | 30500,5715,F10129,F1010,5712,K7031,K7030,5718,K7581,29181,30390,30391,F1021,F10239,30393,F1011,30301,F1020,F10229,30503,5711,30300,K7010,K7040,K7011,K860,30502,2910,K8520,Y908,F10231,I426,G621,F1099,53530,3575,4255,5710,30392,29189,K700,K2920,Y906,K709,K8521,5713,Y907,F10188,F10288,G312,F10230,F10120,K852,30302,F1024,F10929,F10988,2912,F10232,F1014,2913,2911,Y900,K7041,53531,5719,F1094,Y909,Y905,K2921,F10251,F10220,F10221,Y901,Y902,Y904,9809,Y903,F10121,E8600,F1097,Q860,F1027,K8522,K702,F1019,2919,F10151,F1026,E8609,F10920,F10280,F1096,30303,F1029,F10951,O99313,F10259,F10159,T518X2A,O99311,T5192XA,F10959,Z7141,F10180,76071,F10980,E9473,29182,G721,R780,F10250,9773,T5191XA,E244,T5194XA,9808,7903,F10282 |
| Exposure to Other Toxic Substances | 3577,G622,Z575,Z574,T6291XA,T628X1A |
| Encephalopathy | G92,G9341,34982,G9340,34830,5722,34831,34839,G9349,E512,4372,I6783,I674,A812,I67850,I673,J1081,J1181,76870 |
| Liver Failure | K7290,K7200,K7040,K7210,K7201,K7041,K7291,K9182,K7211 |
| Abnormal Renal Function | R944,58889,M1A39X1 |
