# Supplementary figures and images for: Pre-ICU statin therapy reduces 28-day mortality in sepsis-associated brain dysfunction: a propensity-matched analysis of potential neuroprotective mechanisms
Source: Front Pharmacol. 2025 Sep 30;16:1586372. doi: 10.3389/fphar.2025.1586372 (PMC12517583; doi:10.3389/fphar.2025.1586372)

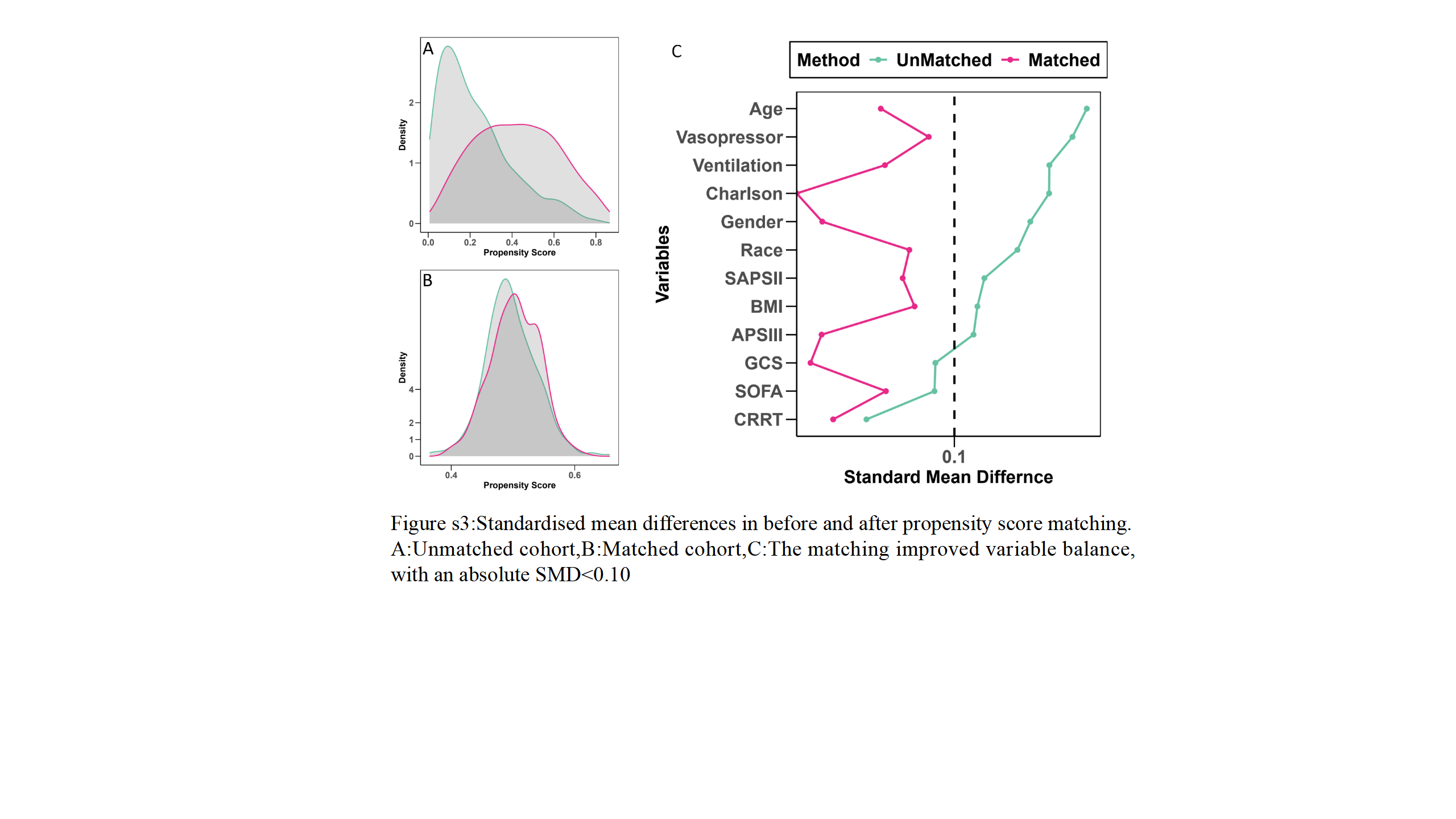

Supplement: Supplementary file 2 [file Image3.tif]

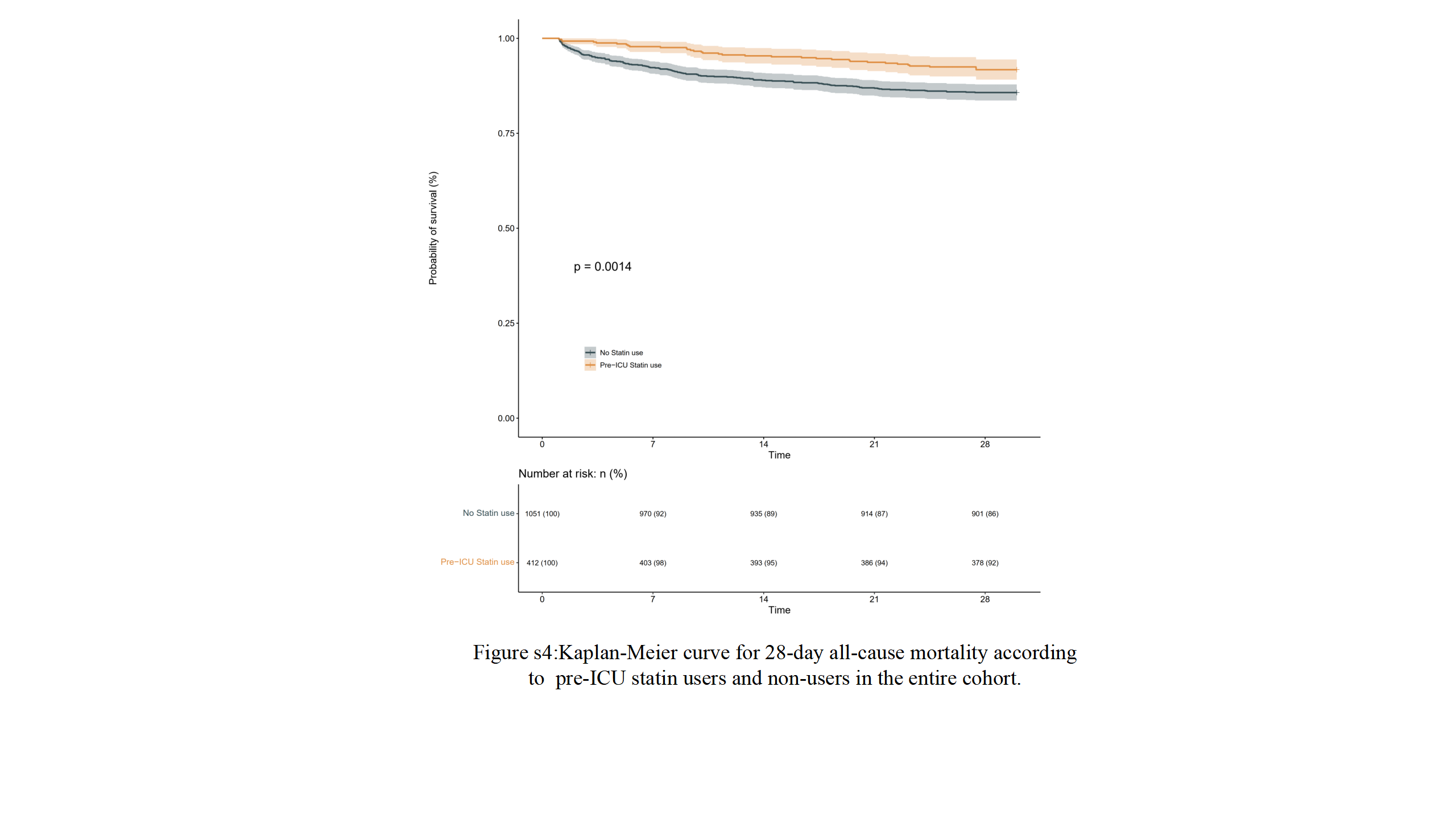

Supplement: Supplementary file 3 [file Image4.tif]

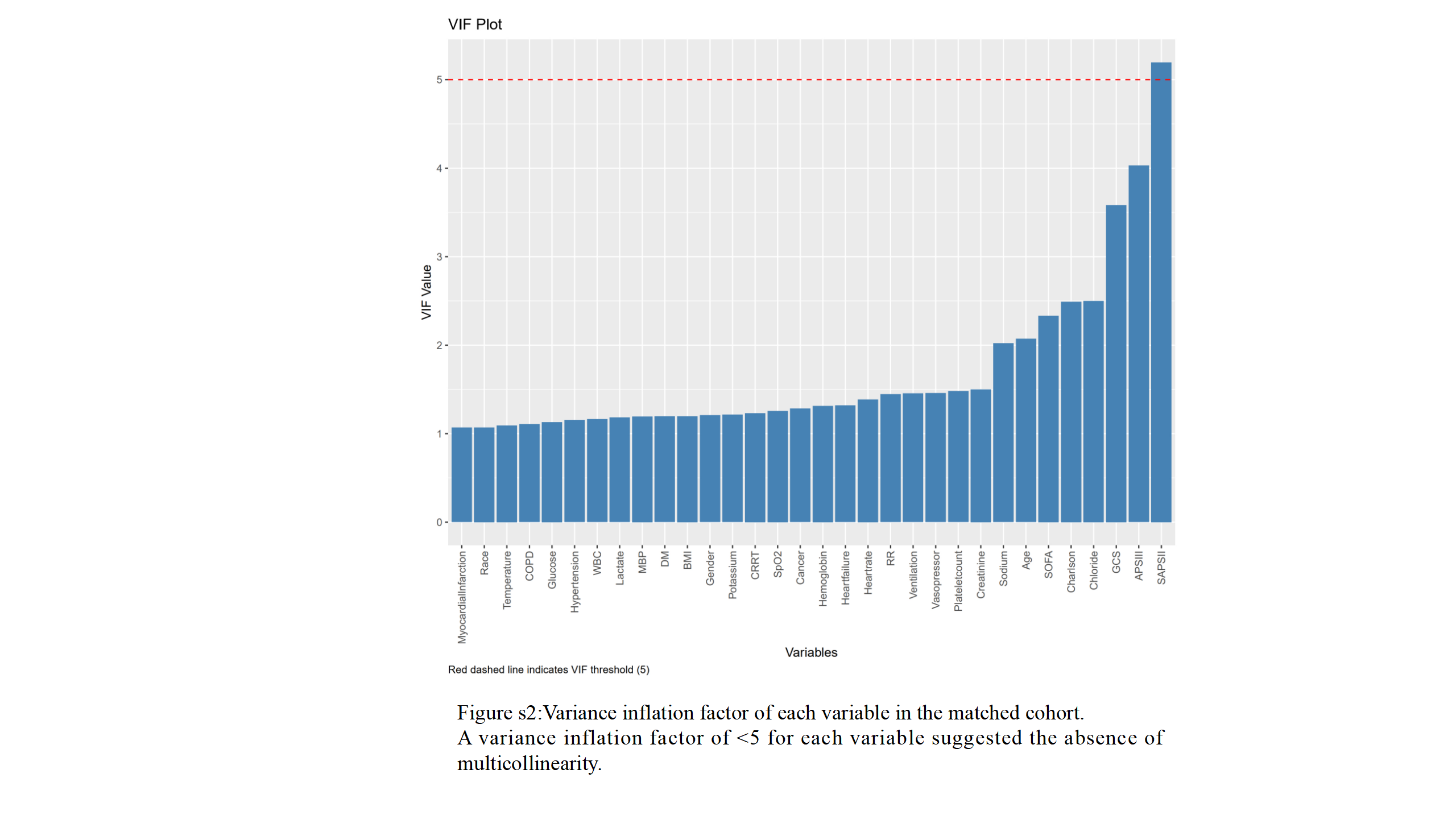

Supplement: Supplementary file 4 [file Image2.tif]

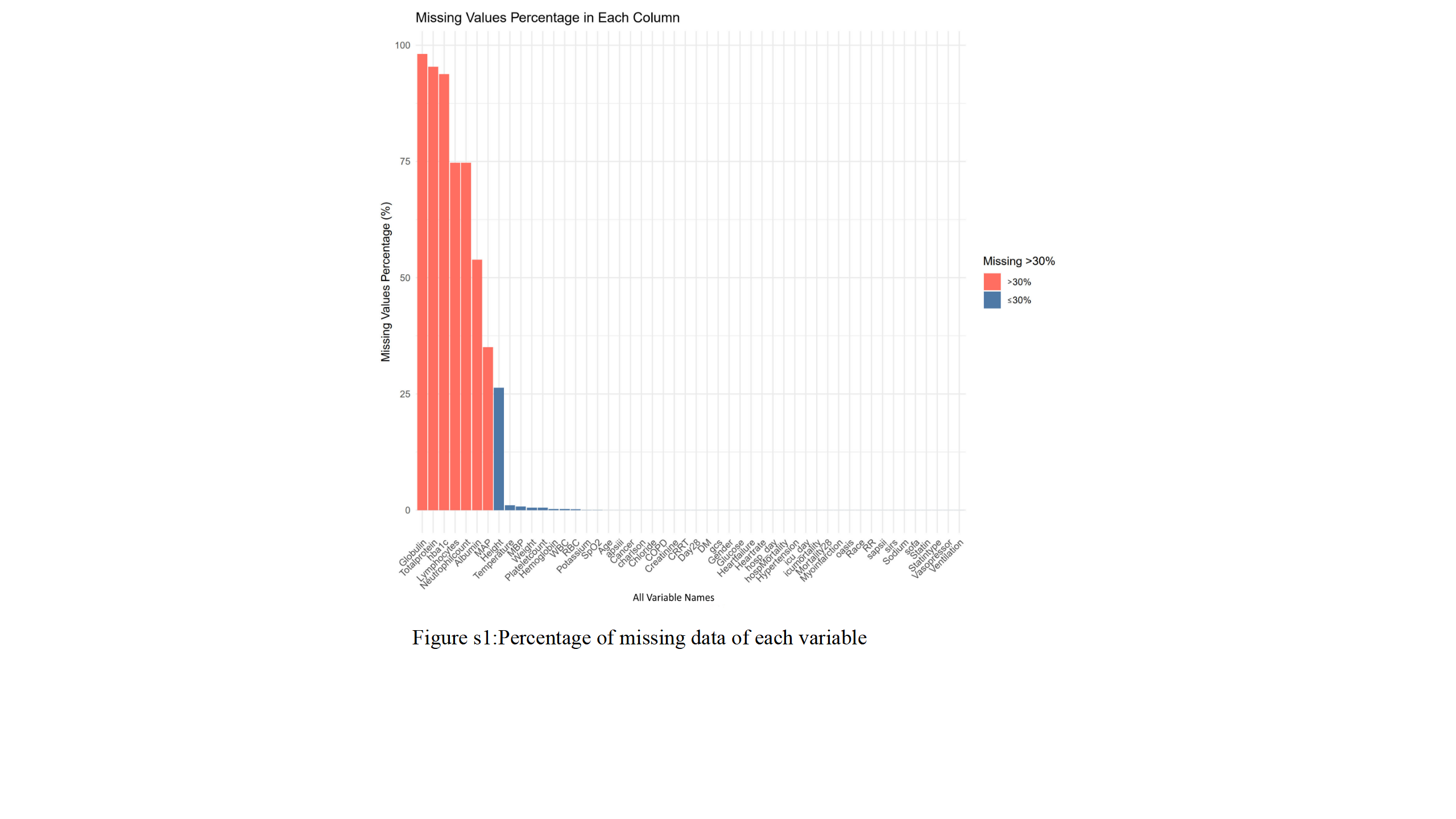

Supplement: Supplementary file 5 [file Image1.tif]
