# Supplementary material for: Pre-ICU statin therapy reduces 28-day mortality in sepsis-associated brain dysfunction: a propensity-matched analysis of potential neuroprotective mechanisms
Source: Front Pharmacol. 2025 Sep 30;16:1586372. doi: 10.3389/fphar.2025.1586372 (PMC12517583; doi:10.3389/fphar.2025.1586372)
Supplement: Supplementary file 6 [file Table2.docx]

***Table 2S：Association Between Pre-ICU Statin Use and Clinical Outcomes in the original cohort***

| **Outcomes** |  | No Statin use （N=1051） | Pre-ICU Statin use （N=412） | Univariable analysis | | Multivariable analysis^※^ | |
| --- | --- | --- | --- | --- | --- | --- | --- |
|  |  |  |  | HR/OR (95% CI) | P-value | HR/OR (95% CI) | P-value |
| **Primary outcome** |  |  |  |  |  |  |  |
| 28-day all-cause mortality^‡^, (n%) |  | 150 (14.3%) | 34 (8.3%) | 0.550(0.379,0.798) | 0.002 | 0.584(0.393,0.867) | 0.008 |
| **Secondary outcomes** |  |  |  |  |  |  |  |
| ICU mortality^†^, n (%) |  | 78 (7.4%) | 14 (3.4%) | 0.439(0.246,0.784) | 0.005 | 0.749(0.275,2.042) | 0.573 |
| In-hospital mortality^†^, n (%) |  | 116 (11.0%) | 25 (6.1%) | 0.521(0.333,0.815) | 0.004 | 0.802(0.367,1.757) | 0.582 |
| Length of ICU stay(days)^†^, median (IQR) |  | 2.99(1.82, 5.70) | 2.21(1.29, 3.95) | 0.977(0.956,0.998) | 0.035 | 1.002(0.982,1.022) | 0.866 |
| Length of hospital stay (days)^†^, median (IQR) |  | 8.45(5.37, 14.99) | 8.11(5.90, 12.40) | 0.996(0.986,1.005) | 0.366 | / | / |

CI, confidence interval; HR, hazard ratio; IQR, interquartile range; OR, odds ratio; ^※^Adjusted for Age, Gender, Race, BMI, Hypertension, Diabetes Mellitus, Malignant tumor, Heart failure, Myocardial Infarction, COPD, Heartrate, MBP, RR, SpO2, Temperature, White Blood Cell count, Platelet count, Hemoglobin, Sodium, Potassium, Chloride, Glucose, Lactate, Creatinine, Vasopressor, Continuous Renal Replacement Therapy, Ventilation, SOFA, APSIII, GCS, and Charlson Comorbidity Index.

^‡^HR with 95% CI was calculated using Cox proportional hazards model.

^†^OR with 95% CI was calculated using logistic regression model.
